# Impacts of Elevated CO_2_ and a Nitrogen Supply on the Growth of Faba Beans (*Vicia faba* L.) and the Nitrogen-Related Soil Bacterial Community

**DOI:** 10.3390/plants13172483

**Published:** 2024-09-05

**Authors:** Xingshui Dong, Hui Lin, Feng Wang, Songmei Shi, Zhihui Wang, Sharifullah Sharifi, Junwei Ma, Xinhua He

**Affiliations:** 1 National Base of International S&T Collaboration on Water Environmental Monitoring and Simulation in the Three Gorges Reservoir Region, Centre of Excellence for Soil Biology, College of Resources and Environment, Southwest University, Chongqing 400715, China; xingshuid@outlook.com (X.D.); 2022020@ynau.edu.cn (S.S.); nsharifullah@gmail.com (S.S.); 2 State Key Laboratory for Managing Biotic and Chemical Threats to the Quality and Safety of Agro-Products, Institute of Environment, Resource, Soil and Fertilizers, Zhejiang Academy of Agricultural Sciences, Hangzhou 310021, China; linhui@zaas.ac.cn (H.L.); wangfeng@zaas.ac.cn (F.W.); 3 State Key Laboratory of Hydraulics and Mountain River Engineering and College of Water Resource and Hydropower, Sichuan University, Chengdu 610065, China; zhihuiwang0928@scu.edu.cn; 4 Department of Land, Air and Water Resources, University of California at Davis, Davis, CA 90616, USA; 5 School of Biological Sciences, University of Western Australia, Perth 6009, Australia

**Keywords:** *Nitrosomonadaceae*, *Nitrososphaeraceae*, redundancy analysis, *Rhizobiaceae*, soil microbial community

## Abstract

Ecosystems that experience elevated CO_2_ (eCO_2_) are crucial interfaces where intricate interactions between plants and microbes occur. This study addressed the impact of eCO_2_ and a N supply on faba bean (*Vicia faba* L.) growth and the soil microbial community in auto-controlled growth chambers. In doing so, two ambient CO_2_ concentrations (aCO_2_, daytime/nighttime = 410/460 ppm; eCO_2_, 550/610 ppm) and two N supplement levels (without a N supply—N0—and 100 mg N as urea per kg of soil—N100) were applied. The results indicated that eCO_2_ mitigated the inhibitory effects of a N deficiency on legume photosynthesis and affected the CO_2_ assimilation efficiency, in addition to causing reduced nodulation. While the N addition counteracted the reductions in the N concentrations across the faba beans’ aboveground and belowground plant tissues under eCO_2_, the CO_2_ concentrations did not significantly alter the soil NH_4_^+^-N or NO_3_^−^-N responses to a N supply. Notably, under both aCO_2_ and eCO_2_, a N supply significantly increased the relative abundance of *Nitrososphaeraceae* and *Nitrosomonadaceae*, while eCO_2_ specifically reduced the *Rhizobiaceae* abundance with no significant changes under aCO_2_. A redundancy analysis (RDA) highlighted that the soil pH (*p* < 0.01) had the most important influence on the soil microbial community. Co-occurrence networks indicated that the eCO_2_ conditions mitigated the impact of a N supply on the reduced structural complexity of the soil microbial communities. These findings suggest that a combination of eCO_2_ and a N supply to crops can provide potential benefits for managing future climate change impacts on crop production.

## 1. Introduction

Atmospheric CO_2_ (aCO_2_) is the primary resource for plant growth and biomass production through photosynthesis. In the face of escalating climate change, the aCO_2_ concentration has been steadily increasing, with an annual rate of 2.0 to 2.4 ppm between 2000 and 2019, reaching a peak of 409.9 ppm in 2019—an upsurge of 5.0% [1]. This trend highlights the acceleration of climate-related concerns, with projections estimating the surge in the CO_2_ concentration to reach 700 ppm by the end of the twenty-first century [1]. Elevated CO_2_ (eCO_2_) concentrations have far-reaching environmental implications, encompassing an escalation in extreme weather phenomena, alterations in pest and disease distributions, and impacts on crop growth and yield stability. Consequently, these factors intensify uncertainties in agricultural production and pose a threat to food security [2,3,4,5,6].

The faba bean (*Vicia faba* L.), with a cultivation history of over 2000 years in China, is a significant leguminous crop that substantially contributes to agricultural production and food safety through its comparatively high productivity and high-quality protein [7]. Studies have found that eCO_2_ enhances the N_2_-fixing capacity of leguminous plants, endowing them with greater productivity than non-N_2_-fixing plants [8,9]. Increased CO_2_ can benefit C_3_ crops such as faba beans by enhancing their photosynthesis, water use efficiency, and yields, particularly under nitrogen (N) fertilization [10]. With a sufficient N supply, plants under eCO_2_ are capable of substantially greater N absorption and experience even less foliar N loss [11]. However, higher N application rates do not necessarily increase faba bean yields at all [12,13].

Soil microorganisms play a crucial role in facilitating nutrient cycling, enhancing plant nutrient absorption, and maintaining soil health through the decomposition of organic matter. An inoculation with N_2_-fixing bacteria can consequently improve N uptake and faba bean growth [14]. In response to environmental changes such as increasing CO_2_ concentrations, soil microorganisms may adapt by modifying their metabolic pathways and community structures, thereby influencing plant growth and soil fertility. It has been observed that eCO_2_ can lead to a significant reduction in soil NO_3_^−^-N, possibly due to increased plant uptake or losses to groundwater and the atmosphere [15,16,17]. Studies have also observed that eCO_2_ significantly increases the relative abundance of genes associated with N_2_ fixation and denitrification in soybean soil, which may be attributed to an increased C input from litter and root exudates [18]. Similarly, in grassland ecosystems, eCO_2_ has been found to stimulate the relative abundance of genes associated with N_2_ fixation. The increase in gene expression has been shown to enhance both the growth and N_2_-fixing rate of leguminous plants [19,20]. When the N availability is high, microbial activity and N utilization are enhanced due to increased CO_2_. These phenomena have significant implications for understanding the impact of eCO_2_ on both C and N cycling in ecosystems [21,22].

Under eCO_2_ conditions, the populations of both ammonia-oxidizing archaea (AOA) and ammonia-oxidizing bacteria (AOB) experience a significant increase, thereby enhancing the nitrification potential of the topsoil [23]. However, eCO_2_ may hinder the denitrification process, potentially disrupting the microbial capacity for intracellular electron transport and utilization [24]. The interaction between eCO_2_ and an anthropogenic N supply also has a significant impact on soil microbial communities and their functional capabilities [25,26,27]. Such interactions may lead to the activation of N-cycling microorganisms in the short term, while previous studies have primarily focused on the taxonomic composition of soil microorganisms or specific functional groups, such as nitrifying bacteria. There remains a limited comprehensive assessment of both the taxonomic and functional aspects of the entire soil microbial community, which is an urgent matter for understanding their relevant functions.

As CO_2_ levels rise, it is imperative to re-evaluate N application rates to update nutrient management strategies for better crop productivity. This will not only ensure that crops benefit from the stimulation of eCO_2_, but will also minimize the loss of grain quality and reduce the risk of N pollution. Faba beans, which fix N through nodulation, can reduce the reliance on chemical N fertilizers. We hypothesized that eCO_2_ and a N supply could (1) increase the tissue N concentration of faba beans; (2) inhibit the nodulation of faba beans; and (3) change the soil microbial community. Current indoor studies are insufficient in considering the comprehensive effects of environmental factors or in-situ field conditions. Therefore, we designed automatically controlled environmental factors to monitor the characteristics of both plants and soil by measuring the changes in plant growth parameters, soil physicochemical properties, and the soil microbial community composition. This study aimed to enhance the yield and quality of faba beans while providing a theoretical basis for a rational N fertilization strategy under rising CO_2_ concentrations. The generated results would then guide agricultural practices to manage soil microorganisms for an effective adaption to climate change, thus promoting agricultural sustainability.

## 2. Results

### 2.1. Variation in Plant Growth Characteristics

**Figure 1:**
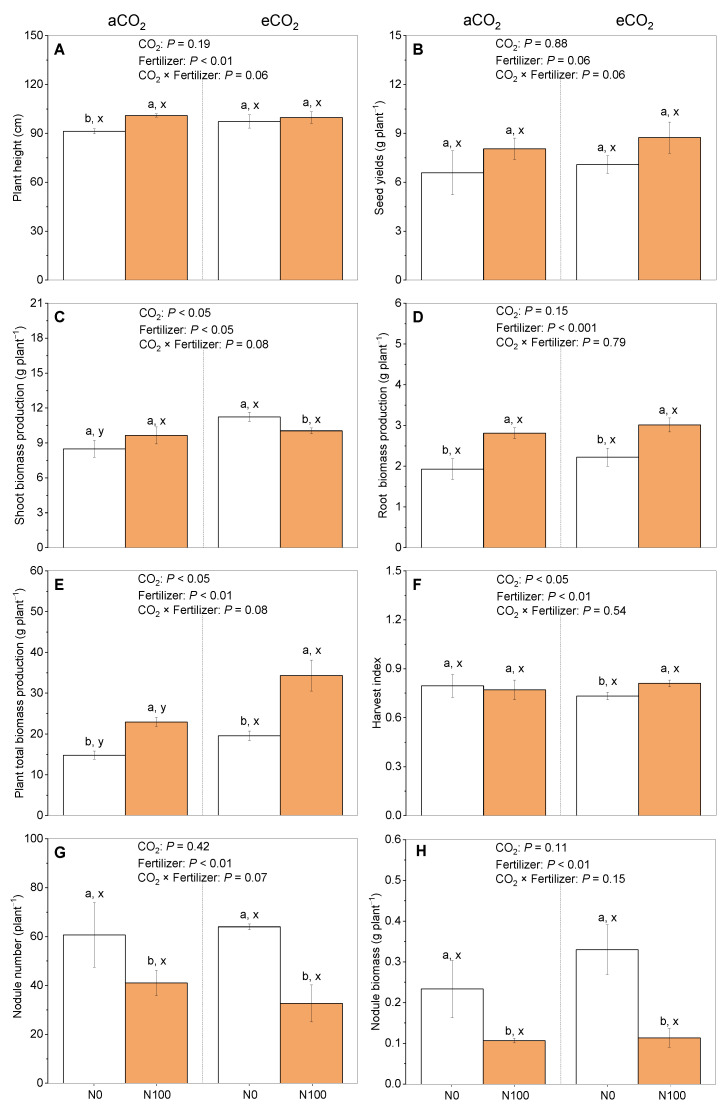
The effects of CO_2_ and a N supply on 5-month-old faba beans at harvest. (**A**) Plant height; (**B**) seed yields; (**C**) shoot biomass production (stem and leaf); (**D**) root biomass production (root and nodules); (**E**) plant total biomass production; (**F**) harvest index = seeds/shoot biomass; (**G**) nodule number; and (**H**) nodule biomass. The data are the means ± SE (*n* = 3). Lower-case letters above the bars indicate significant differences between N supply levels for the same CO_2_ treatment (a, b) and between CO_2_ concentrations for the same N treatment (x, y) at *p <* 0.05. The results of the two-way ANOVA are also presented at different *p* levels to show the interactive effects of CO_2_ × N supply on the measured variables. Abbreviations: aCO_2_, atmospheric CO_2_; eCO_2_, elevated CO_2_; N0, no N supply; N100, 100 mg N kg^−1^ DW soil.

Under aCO_2_, a N supply to 5-month-old faba beans significantly increased the plant height and the root and plant total biomass production (Figure 1A,B,E), with no impact on the seed yields, shoot biomass, or harvest index (Figure 1B,C,F), but it significantly decreased the number and biomass production of nodules (Figure 1G,H). In contrast, under eCO_2_, a N supply significantly increased the root and plant total biomass production and the harvest index (Figure 1D–F), with no impact on the plant height or seed yields (Figure 1A,B) but it had a negative impact on shoot biomass production and the number and biomass of nodules (Figure 1C,G,H). Interestingly, a significantly interactive effect of eCO_2_ and a N supply on the plant total biomass production was shown under both N0 and N100, i.e., irrespective of a N supply (Figure 1E), but was shown for shoot biomass production under N0 only (Figure 1C). These results indicate that eCO_2_ generally enhanced the positive effects of N on faba bean growth while having an inhibitory effect on faba bean nodule formation.

### 2.2. Variation in Basic Photosynthetic Characteristics

**Figure 2:**
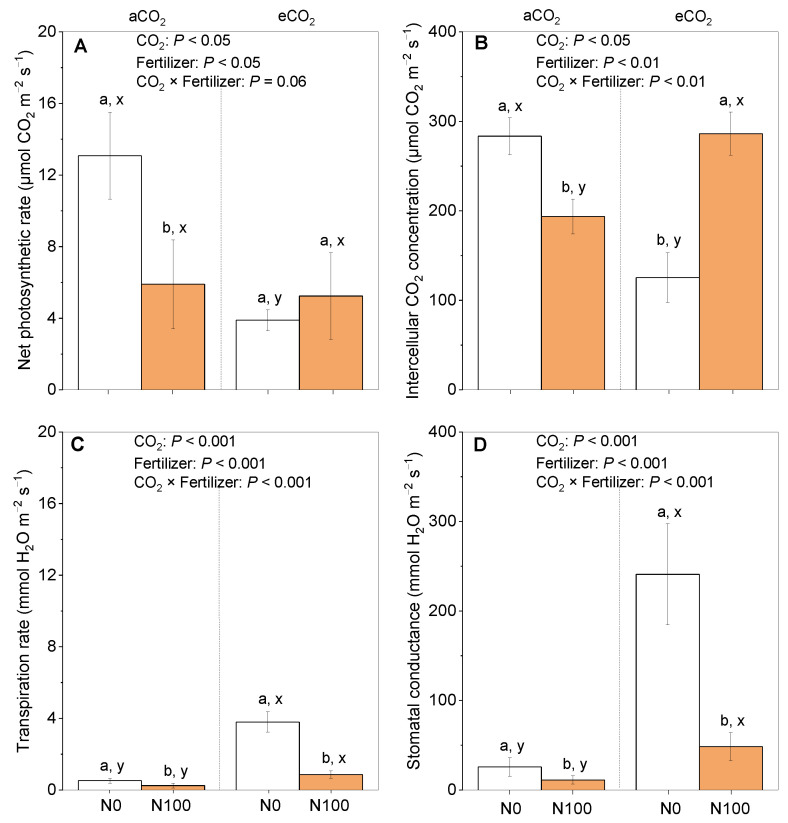
The effects of CO_2_ and a N supply on photosynthesis parameters during the flowering stage of faba beans. (**A**) Net photosynthetic rate; (**B**) intercellular carbon dioxide concentration; (**C**) transpiration rate; and (**D**) stomatal conductance. The data are the means ± SE (*n* = 3). Lower-case letters above the bars indicate significant differences between N supply levels for the same CO_2_ treatment (a, b) and between CO_2_ concentrations for the same N treatment (x, y) at *p <* 0.05. The results of the two-way ANOVA are also presented at different *p* levels to show the interactive effects of CO_2_ × N supply on the measured variables. Abbreviations: aCO_2_, atmospheric CO_2_; eCO_2_, elevated CO_2_; N0, no N supply; N100, 100 mg N kg^−1^ DW soil.

The effects of CO_2_ levels and N on photosynthesis parameters during the flowering stage of faba beans showed diverse differences. An application of N fertilizer significantly decreased the net photosynthetic rate (Figure 2A), intercellular CO_2_ concentration (Figure 2B), transpiration rate (Figure 2C), and stomatal conductance (Figure 2D) under aCO_2_, while significantly increasing the intercellular CO_2_ concentration had no impact on the net photosynthetic rate, but decreased both the transpiration rate and stomatal conductance under eCO_2_. In addition, at N0, the net photosynthetic rate and intercellular CO_2_ concentration were significantly greater under aCO_2_ than under eCO_2_ (Figure 2A vs. Figure 2B), but the opposite was true for the transpiration rate and stomatal conductance under aCO_2_ than under eCO_2_ (Figure 2A vs. Figure 2B). At N100, the tested parameters, including the intercellular CO_2_ concentrations, transpiration rate, and stomatal conductance, were significantly greater under eCO_2_ than under aCO_2_.

### 2.3. Variation in Tissue N Concentrations

**Figure 3:**
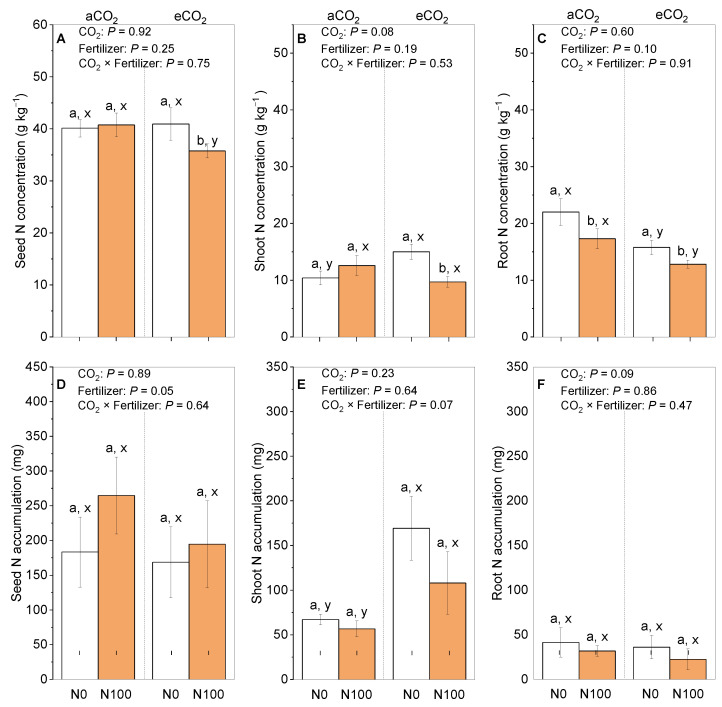
The effects of CO_2_ and a N supply on the tissue N concentrations and accumulations in seeds (**A**,**D**), shoots (stems and leaves) (**B**,**E**), and roots (**C**,**F**) of 5-month-old faba beans at harvest. The data are the means ± SE (*n* = 3). Lower-case letters above the bars indicate significant differences between N supply levels for the same CO_2_ treatment (a, b) and between CO_2_ treatments for the same N treatment (x, y) at *p <* 0.05. The results of the two-way ANOVA are also presented at different *p* levels to show the interactive effects of CO_2_ × N supply on the measured variables. Abbreviations: aCO_2_, atmospheric CO_2_; eCO_2_, elevated CO_2_; N0, no N supply; N100, 100 mg N kg^−1^ DW soil.

Under aCO_2_, N100 had an insignificant influence on the seed and shoot N concentrations, which were, respectively, 2% and 10% higher than under N0 (Figure 3A,B), but N100 significantly decreased the root N concentrations (Figure 3C). Compared to N0, the N accumulation under a N supply increased by 4% in the seeds, decreased by 4% in the roots, and did not change at all in the shoots under aCO_2_ (Figure 3D). In contrast, under eCO_2_, a N supply significantly decreased the N concentrations in the shoots, roots, and seeds. Under eCO_2_, the N accumulation under a N supply greatly increased by 16% in the seeds and decreased by 6% in the shoots, but no changes were observed at all in the roots (Figure 3E,F). Additionally, eCO_2_ significantly increased the shoot N concentrations and seed N concentrations in the N0 treatment. However, eCO_2_ significantly decreased the root N concentrations under both the N0 and N100 treatments and the shoot N concentrations under the N100 treatment (Figure 3B,C).

### 2.4. Changes in Soil pH, Organic Matter, and Concentrations of NH_4_^+^-N and NO_3_^−^-N

**Figure 4:**
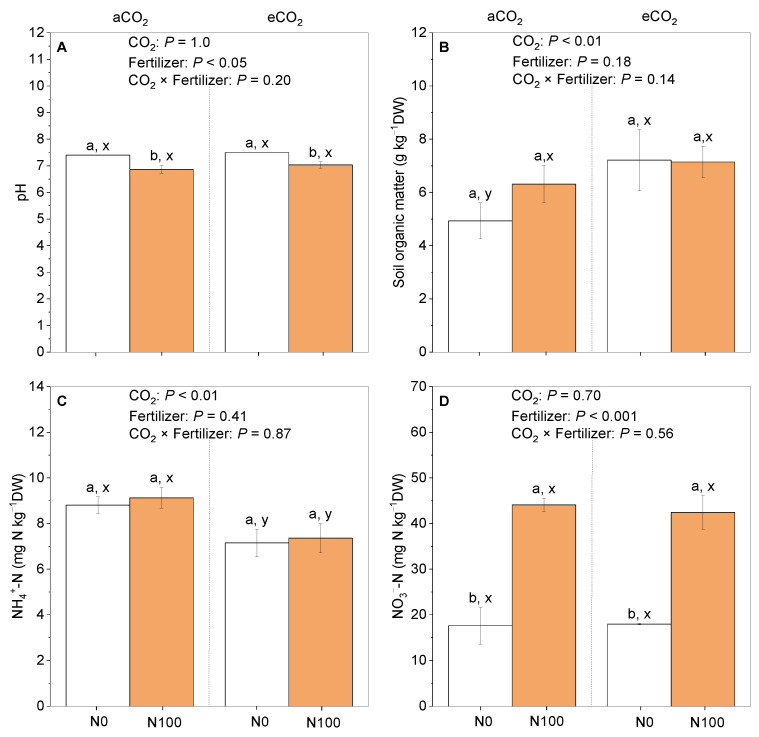
The effects of CO_2_ and a N supply on (**A**) soil pH; (**B**) soil organic matter; (**C**) NH_4_^+^-N; and (**D**) NO_3_^−^-N at the harvest of 5-month-old faba beans. The data are the means ± SE (*n* = 3). Lower-case letters above the bars indicate significant differences between N supply levels for the same CO_2_ treatment (a, b) and between CO_2_ concentrations for the same N treatment (x, y) at *p <* 0.05. The results of the two-way ANOVA are also presented at different *p* levels to show the interactive effects of CO_2_ × N supply on the measured variables. Abbreviations: aCO_2_, atmospheric CO_2_; eCO_2_, elevated CO_2_; N0, no N supply; N100, 100 mg N kg^−1^ DW soil.

Under aCO_2_ or eCO_2_, a N supply resulted in a significant decrease in the soil pH (Figure 4A) and no changes in the soil organic matter or soil NH_4_^+^-N concentrations (Figure 4C), while a significant increase in the soil NO_3_^−^-N concentrations was observed (Figure 4D). In contrast, irrespective of the N supply, the soil NH_4_^+^-N concentrations were significantly lower under eCO_2_ than under aCO_2_ (Figure 4C). Overall, the soil pH was decreased by a N supply, while the soil NH_4_^+^-N was decreased by eCO_2_.

### 2.5. Relationships between Soil Inorganic N and Plant N Characteristics

**Figure 5:**
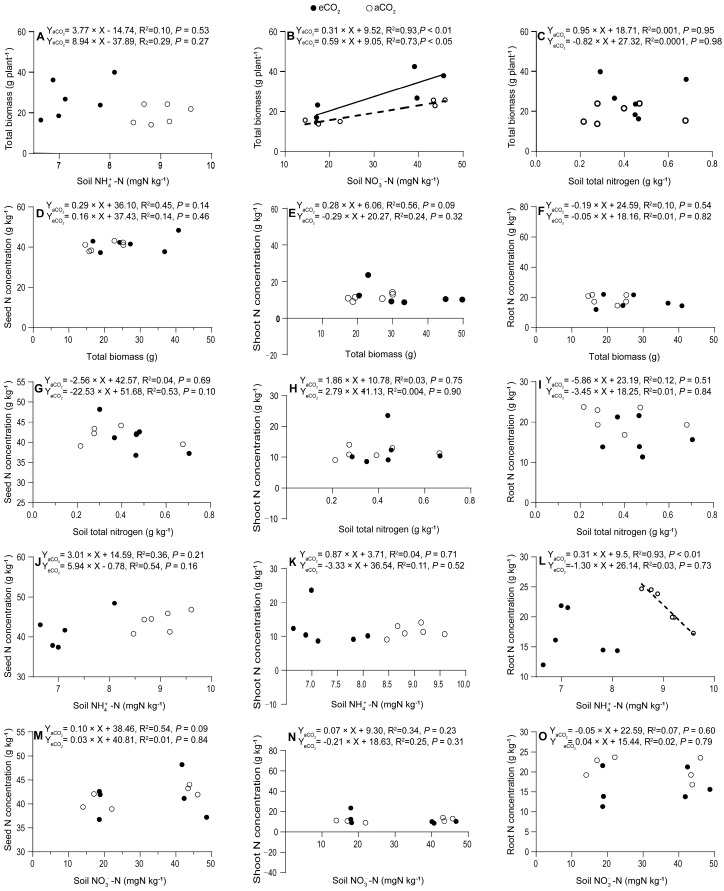
Relationships between soil NH_4_^+^-N (**A**), soil NO_3_^−^-N (**B**) or soil total nitrogen (**C**) and plant total biomass production (**A**–**C**); between tissue N concentrations in seed (**D**), shoot (**E**) or root (**F**) and plant total biomass production (**D**–**F**); between tissue N concentrations in seed (**G**), shoot (**H**) or root (**I**) and soil total nitrogen (**G**–**I**); between tissue N concentrations in seed (**J**), shoot (**K**) or root (**L**) and soil NH_4_^+^-N (**J**–**L**); between tissue N concentrations in seed (**M**), shoot (**N**) or root (**O**) and soil NO_3_^−^-N (**M**–**O**) in 5-month-old faba beans grown under atmospheric CO_2_ (aCO_2_) and elevated CO_2_ (eCO_2_). The open and closed circles represent data under aCO_2_ and eCO_2_, respectively. Regressions are shown for the aCO_2_ (dotted lines) and eCO_2_ (solid lines) treatments; *n* = 6.

The faba bean total biomass production did not show correlations with the soil NH_4_^+^-N (*p* = 0.27–0.53, Figure 5A), soil total N concentrations, or plant tissue total N concentrations (*p =* 0.95–0.98, Figure 5C), while it was significantly positively correlated with the soil NO_3_^−^-N under both aCO_2_ (*p <* 0.01) and eCO_2_ (*p <* 0.05) (Figure 5B). The nitrogen concentrations in faba bean tissues, including the seeds, shoots, and roots, had no correlations with the total biomass production (*p =* 0.09–0.82, Figure 5D–F). No correlations were observed between the seed N concentrations, shoot N concentrations, or root N concentrations and the soil total N (*p =* 0.10–0.90, Figure 5G–I), soil NH_4_^+^-N (*p =* 0.16–0.73, Figure 5J–L), or soil NO_3_^−^-N (*p =* 0.09–0.84, Figure 5M–O) under either aCO_2_ or eCO_2_, except for a positive correlation between the root N concentration and the soil NH_4_^+^-N concentrations under aCO_2_ (*p =* 0.01, Figure 5L).

**Figure 6:**
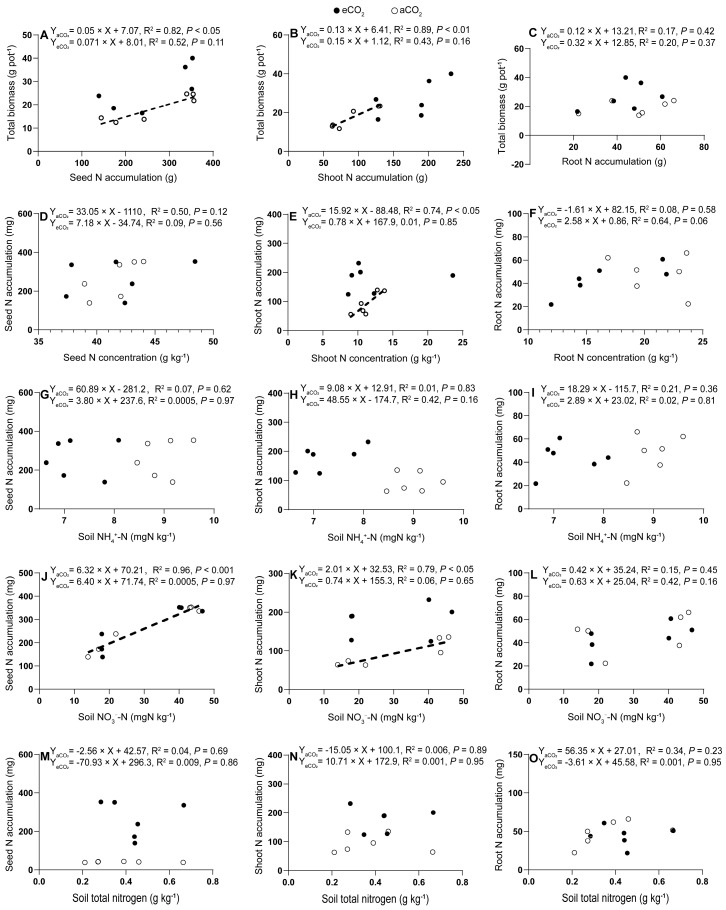
Relationships between tissue N accumulations in seed (**A**), shoot (**B**) or root (**C**) and plant total biomass production (**A**–**C**); between tissue N concentrations in seed (**D**), shoot (**E**) or root (**F**) and tissue N accumulations in seed (**D**), shoot (**E**) or root (**F**); between tissue N accumulations in seed (**G**), shoot (**H**) or root (**I**) and soil NH_4_^+^-N (**G**–**I**); between tissue N accumulations in seed (**J**), shoot (**K**) or root (**L**) and soil NO_3_^−^-N (**J**–**L**); between tissue N accumulations in seed (**M**), shoot (**N**) or root (**O**) and soil total nitrogen (**M**–**O**) in 5-month-old faba beans grown under atmospheric CO_2_ (aCO_2_) and elevated CO_2_ (eCO_2_). The open and closed circles represent data under aCO_2_ and eCO_2_, respectively. Regressions are shown for the aCO_2_ (dotted lines) and eCO_2_ (solid lines, there is no solid regression line because of no correlation in the eCO_2_) treatments; *n* = 6.

The faba bean total biomass production was correlated with the seed N and shoot N accumulations only under aCO_2_ (*p* < 0.05, Figure 6A; *p* < 0.01, Figure 6B), but not with the root N accumulations under either aCO_2_ or eCO_2_ (*p* = 0.37–0.42, Figure 6C). However, the seed and root N accumulations did not correlate with the seed and root N concentrations under either aCO_2_ or eCO_2_ (*p =* 0.12–0.58, Figure 6D,F), except for a positive correlation between the shoot N concentration and the shoot N accumulation under aCO_2_ (*p =* 0.01, Figure 6E). Nitrogen accumulations in faba bean tissues, including the seeds, shoots, and roots, did not show correlations with the soil NH_4_^+^-N (*p =* 0.16–0.97, Figure 6G–I), soil NO_3_^−^-N (*p =* 0.16–0.97, Figure 6J–L), or soil total N (*p =* 0.23–0.95, Figure 6M–O) under either aCO_2_ or eCO_2_, except for a positive correlation between the seed and root N accumulations and the soil NO_3_^−^-N concentrations under aCO_2_ (*p <* 0.05–0.01, Figure 6J,K).

### 2.6. Variation in Soil Microbial Community and Structure

The impact of eCO_2_ and a N supply on soil microbial communities was assessed using high-throughput sequencing of the total 16S rRNA genes. A total of 9,925,182 valid sequences were obtained from the soil sample, with individual sample sequence counts ranging from 33,864 to 143,936 and an average of 63,622 sequences per sample. The sequences belonged to 50 microbial phyla, with 89.6% belonging to eight bacterial phyla (*Actinobacteriota*, *Bacteroidota*, *Chloroflexi*, *Firmicutes*, *Myxococcota*, *Patescibactera*, *Planctomycota*, and *Proteobacteria*).

**Figure 7:**
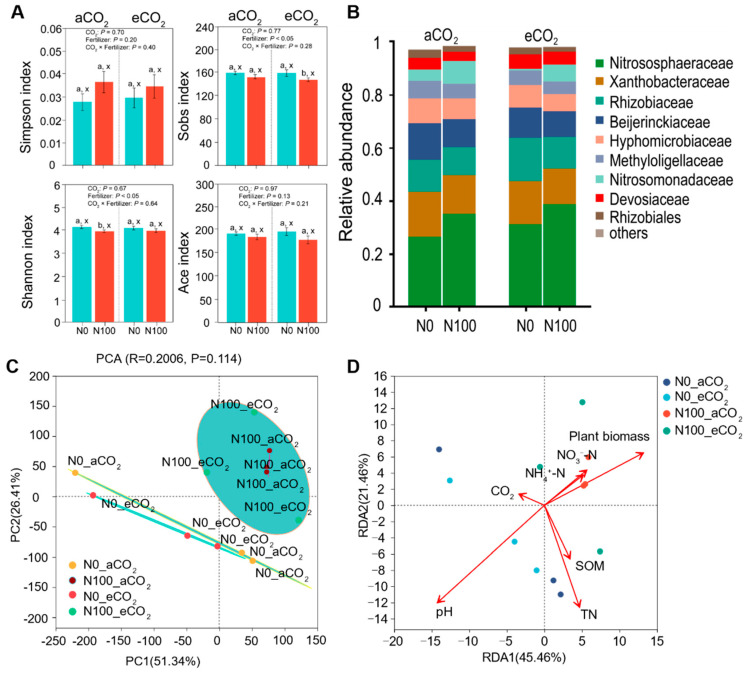
Transitions of soil microbes under different CO_2_ levels and N supply levels. (**A**) Simpson’s, Shannon’s, Sob’s, and Ace’s microbial diversity indices at the OTU level. The data are the means ± SE (*n* = 3). Lower-case letters above the bars indicate significant differences between different N supply treatments for the same CO_2_ treatment (a, b) and between different CO_2_ concentrations for the same N treatment (x, y) at *p <* 0.05. The results of the two-way ANOVA are also presented at different *p* levels to show the interactive effects of CO_2_ × N supply on the measured variables. (**B**) The relative abundance of the microbial community at the family level. (**C**) A principal component analysis (PCA) of the microbial community composition at the OTU level. (**D**) A redundancy analysis (RDA) plot showing the relationship between environmental factors and the microbial community structure at the OTU level. Abbreviations: aCO_2_, atmospheric CO_2_; eCO_2_, elevated CO_2_; N0, no N supply; N100, 100 mg N kg^−1^ DW soil.

Under aCO_2_, a N supply significantly decreased the Shannon index. Under eCO_2_, a N supply significantly lowered the Sobs index, but had little impact on Simpson’s index or the Ace index (Figure 7A). Under aCO_2_, a N supply notably increased the relative abundance of *Nitrososphaeraceae* and *Nitrosomonadaceae* at the family level; this trend was similar under eCO_2_. However, a N supply decreased *Rhizobiaceae* under eCO_2_ (Figure 7B).

**Figure 8:**
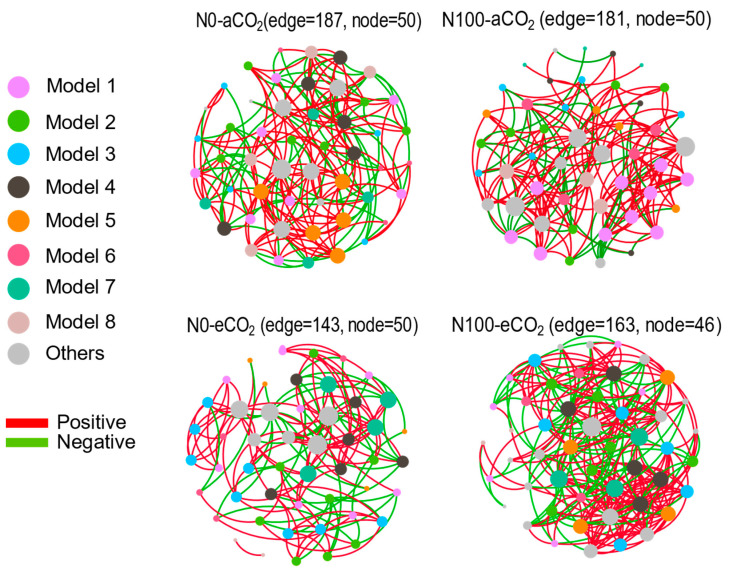
Microbial co-occurrence networks were constructed for no N supply (N0) and a N supply (N100), and for soil exposed to atmospheric CO_2_ (aCO_2_) and elevated CO_2_ (eCO_2_). Each network was further divided into sub-networks, referred to as modules, which contained a set of microbes with numerous interactions. The layout and taxonomic profiles of eight domain modules (M1–M8) were analyzed. Nodes in the network represent microbes, while edges represent statistically significant associations between nodes. Abbreviations: aCO_2_, atmospheric CO_2_; eCO_2_, elevated CO_2_; N0, no N supply; N100, 100 mg N kg^−1^ DW soil.

A PCA showed that the CO_2_ treatment insignificantly influenced N-cycling microorganisms (*p* = 0.114, Figure 7C). In contrast, a N supply highly changed the community structure of N-cycling microorganisms. To determine whether environmental characteristics had an additional effect on the community structure, we performed a redundancy analysis (RDA), a constrained ordination technique that attempts to explain differences in the microbial composition between samples by differences in explanatory variables (e.g., disease status). In the RDA (RDA1 = 45.46%, RDA2 = 21.46%), the soil pH was the most influential factor (*p* < 0.01) on soil ammonia-oxidizing microbes (Figure 7D). These factors collectively determined the distribution pattern of soil N-cycling microbial communities. Co-occurrence networks were applied to reveal the complexity of connections among soil microbial communities (Figure 8 and Supplementary Table S1). Under aCO_2_ and eCO_2_, a N supply decreased the negative edges and increased the positive edges. Under N0 and N100, eCO_2_ decreased the negative edges and increased the positive edges. Under eCO_2_ conditions, a N supply increased the average degree, the diameter graph density, the average clustering coefficient, and the average path length. Under eCO_2_, the impact of a N supply on the dynamics of direct and indirect interactions among species within the microbial community was found to be diminished. These results suggested that a microbial community could maintain a highly connected and resilient interaction network, despite variations in N availability under a high CO_2_ level. These findings highlight how eCO_2_ concentrations can offset the effect of a N supply on the decreased abundance of soil microbial communities.

## 3. Discussion

### 3.1. eCO_2_ and N Supply Enhanced the Biomass Production of Fababean

The faba bean biomass production was significantly increased by elevated CO_2_ (eCO_2_) and a N100 supply (Figure 1D,E), which is supported by other studies [28,29,30]. An adequate N supply is crucial for faba bean growth and development (Figure 5B) [31]. eCO_2_ could further enhance plant growth by increasing the availability of carbon skeletons for the synthesis of essential biomolecules [30]. This combined effect can lead to increased faba bean biomass production. In contrast, the faba bean nodule biomass was reduced by a N supply under both aCO_2_ and eCO_2_ (Figure 1G,H and Figure 5L), which is the same result as that found in a previous study indicating that high rates of N fertilization could inhibit the formation of root nodules or reduce their effectiveness [12,32]. This means that the readily available N from fertilizer remains an important N source for faba beans, which may rely on it to a considerable extent compared to the N derived from their symbiotic relationship with N-fixing bacteria.

### 3.2. eCO_2_ Offset the Inhibition of a N Supply on Photosynthetic Parameters

Nitrogen plays a crucial role in plant photosynthesis, and the supply status of N directly or indirectly affects the efficiency of photosynthesis and the plant growth performance [33]. A nitrogen application under aCO_2_ usually inhibits the net photosynthetic rate of legume crops [34]. We observed a similar inhibition by a N supply on the net photosynthesis and CO_2_ assimilation (Figure 2). The application of N may impact the content and stability of leaf chlorophyll, as well as the opening and closing of stomata, thereby affecting gas exchange and photosynthesis [35,36]. Meanwhile, excessive N application has previously been found to stimulate rapid plant growth, leading to an imbalance between vegetative and reproductive growth [37]. This imbalance ultimately affects the distribution and utilization of photosynthetic products, resulting in a decrease in the net photosynthetic rate [37].

Our findings suggest that the presence of eCO_2_ could mitigate the adverse consequences of N limitation (Figure 2). This mitigation can be attributed to the high concentration of CO_2_, which facilitates improved carbon assimilation. This study indicates that the presence of eCO_2_ enhances the positive impact of a N supply on the harvest index of faba beans and the suppression of the photorespiration pathway [38]. Collectively, these observations suggest that eCO_2_ levels positively impact photosynthesis and CO_2_ assimilation under N100 (Figure 2). These suppositions were based on the photosynthetic parameters measured at a single sampling time during the flowering stage; these parameters can vary dynamically as the plant progresses through different growth stages. At certain stages, such as flowering, the photosynthetic rate might be higher, but the products are primarily used for the development of reproductive organs rather than increasing the overall biomass [39]. This allocation of resources may lead to a negative relationship between the total biomass production at harvest (Figure 1E) and the net photosynthetic rate observed during the flowering stage (Figure 2A). When the stomatal conductance was increased by eCO_2_, the intercellular carbon dioxide concentration did not rise, but the transpiration rate did (Figure 2). If the transpiration rate is excessively high (usually under conditions of high temperatures and low humidity), plants may close their stomata to reduce water loss [40]. After the stomata close, the entry of carbon dioxide is also limited, leading to a decrease in the intercellular carbon dioxide concentration [40]. In this situation, the stomatal conductance would be extremely high, while the net photosynthetic rate would not be able to respond to such stomatal conductance changes well.

### 3.3. eCO_2_ and N Supply Reduced Root N, but Increased Seed N

The concentrations of N in both the shoots and roots of faba beans were reduced under eCO_2_ (Figure 3A–C and Figure 6K). These findings align with previous studies that have reported similar decreases in the N concentrations in the shoots and roots of faba beans under eCO_2_ levels ranging from 550 to 800 ppm [41,42,43,44]. Nonetheless, it is important to note that, despite this reduction, the overall biomass of the plant and its interactions with rhizobia were enhanced, leading to an overall increase in the seed N (Figure 1E, Figure 3D and Figure 5B). The decline in the N concentration in faba bean plants under eCO_2_ can be attributed to several underlying mechanisms. Firstly, the dilution effect resulting from the increased biomass plays a significant role in this reduction [45,46]. Additionally, decreased transpiration rates, which limit nutrient uptake, contribute to the overall decrease in N rates [42,47,48]. Furthermore, the reduced levels of the Rubisco enzyme, a key player in photosynthesis, further exacerbate the situation by hindering N assimilation [49,50]. The diminished dark respiration under eCO_2_ may result in a decrease in energy-rich compounds in the cytoplasm [51]. This reduction in energy availability could lead to a scarcity of the reductants required for N reduction, thereby impacting the soil N absorption process [52,53].

The results also indicated that a N supply played a supplementary role against the decrease in the seed N concentrations caused by eCO_2_ (Figure 3), which is supported by other studies. The effect of elevated CO_2_ on the potential denitrification of soils and data on the soil available N are presented, which may be related to the complementary effect of a N supply on the seed N concentration [54]. The physiological responses of crops to an increased atmospheric CO_2_ concentration, including the effects of N fertilizers on the plant leaf N concentration, may be related to the effect of a N supply under eCO_2_ conditions [55]. This highlights the importance of nutrient management strategies to maintain proper N uptake and distribution in faba bean plants as the aCO_2_ levels continue to rise.

### 3.4. N Supply Increased Soil NO_3_^−^-N Concentration While Decreasing Soil pH

Nitrification is a central component of the soil N cycle and is responsible for the oxidation of NH_4_^+^ to NO_3_^−^, a key nutrient source for plant growth [56]. Changes in the soil pH directly influence the growth and activity of nitrifying bacteria and ammonia-oxidizing bacteria, thereby affecting the rate and efficiency of nitrification [57]. In alkaline soils, nitrifying bacteria are more active, while acidic soils favor the dominance of ammonia-oxidizing bacteria [58]. Soil acidification following the use of N fertilizers is attributed to the process of ammonium nitrification, wherein each mole of NH_4_^+^ undergoing nitrification releases two hydrogen atoms [59]. Under both eCO_2_ and aCO_2_, the soil pH became more acidic upon N application, and a N supply led to a significant increase in the NO_3_^−^ levels (Figure 4). The level of soil N is also a significant factor affecting the structure and function of nitrifying communities [60]. Higher levels of soil available N, indicating a richer N source, can enhance the abundance and activity of nitrifying bacteria. The genera that are particularly sensitive to changes in the N supply include *Nitrospira*, which shows a notable response to variations in N levels. In contrast, *Devosia*, which is crucial for nodule formation and N_2_ fixation, may be affected by insufficient levels of available N; thus, the available N can restrict the growth and functionality of these bacteria [60,61,62].

### 3.5. N Supply Increased the Relative Abundance and Structural Complexity of Nitrososphaeraceae

Plants absorb N primarily as NH_4_^+^, NO_3_^−^, and some organic N compounds. Microbes such as rhizobia, nitrifying bacteria, denitrifying bacteria, and mycorrhizal fungi facilitate this process [63]. The plant biomass showed a significant correlation with the soil NO_3_^−^-N under both the N0 and N100 conditions (Figure 5B), but no significant correlation was found with the soil NH_4_^+^-N (Figure 5A). NO_3_^−^-N is more readily absorbed and utilized by faba beans compared to NH_4_^+^-N and it exhibits a greater stability in soil, making it less susceptible to changes in the soil pH [64]. Soil microorganisms convert NH_4_^+^-N to NO_3_^−^-N through nitrification, enabling its absorption by faba beans [65]. Additionally, NO_3_^−^-N shows a stronger correlation with the soil microbial community (Figure 7C,D). Under aCO_2_ and eCO_2_, N100 boosted the growth of *Nitrososphaeraceae* and *Nitrosomonadaceae*, bacteria that play a key role in nitrification (Figure 7B). At the same time, it reduced the abundance of *Rhizobiaceae*, which are responsible for nitrogen fixation (Figure 7B). Applying the right amount of N can improve faba beans’ overall health [32]. If faba beans obtain chemical nitrogen from fertilizers, they might not need as much help from the nodules to fix nitrogen, and this can slow down the nitrogen-fixing process [66].

A previous study elucidated that eCO_2_ significantly augments the abundance of genes related to N_2_ fixation, ammonification, denitrification, and assimilatory N reduction at both the 0–5 cm and 5–15 cm soil depths [67]. This observed enhancement can be ascribed to the profound impact of eCO_2_ on soil microbial communities. Such an influence could be mediated through the following mechanisms: an increase in the C input from plants, alterations in the quality of plant litter (including changes in the carbon and N percentages), and modifications in soil characteristics (encompassing different pH and moisture levels) [25,68,69,70]. In this study, the PCA showed that a N supply greatly changed the community structure of soil ammonia-oxidizing microbes, and the RDA indicated that the soil pH was the most influential factor on soil ammonia-oxidizing microbes (Figure 7). The effectiveness of rhizobia, which are known to facilitate a plant’s N acquisition, appears to be compromised under eCO_2_ conditions [71]. This diminished efficacy could be attributed to alterations in the plant’s nutrient requirements and a general decrease in both energy and nutrient demands [72,73].

### 3.6. eCO_2_ Decreased the Abundance of Microorganism and a N Supply Increased the Structural Complexity of Microbial Communities

By utilizing 16S rRNA gene sequencing, our findings indicated that a N supply markedly increased the structure and the abundance of certain bacterial families involved in nitrification and N_2_ fixation processes (Figure 7). Within microbial community networks, under conditions of eCO_2_, a N supply fostered more intricate interactions among microbial entities (Figure 8). Under aCO_2_ and eCO_2_, a N supply decreased the negative edges (competition) and increased the positive edges (cooperation). Under N0 and N100, eCO_2_ decreased the negative edges (competition) and increased the positive edges (cooperation). Such shifts could result in alterations in a community’s resilience and resistance to disturbances, thereby impacting its long-term stability and biodiversity [74]. Several reasons may contribute to the subtle difference in microbial responses to a N supply under eCO_2_. First, the composition and functional structure of microbial communities were significantly different between the two N supply levels, thus resulting in a differential functional potential/activity [75,76,77,78]. Second, the soil may contain more organic matter from plant residues under eCO_2_, resulting in differences in nutrient availability for microbial growth and activities [79,80,81,82,83]. Third, the soil physiochemical properties (e.g., soil aggregate size, pH, temperature, moisture, etc.) may change with the N supply and some of them (e.g., temperature, soil moisture) may experience wider fluctuations under eCO_2_ than under aCO_2_, thus differentially affecting the microbial responses to a N supply under eCO_2_ [84,85,86,87].

## 4. Materials and Methods

### 4.1. Description of Experimental Site

The experimental site was in the National Monitoring Base of Purple Soil Fertility and Fertilizer Effect (29°48′ N, 106°24′ E, 266.3 m above sea level) on the campus of Southwest University, Beibei District, Chongqing, China, located in the purple hilly region with a subtropical monsoon climate. The soil used in this study was purple soil (Eutric Regosol, the FAO Soil Classification System), which developed from the purple mud and shale of the Jurassic Shaximiao Formation [88]. Its basic chemical properties were as follows: a pH of 7.4 (1:2.5 *w*/*v*, soil/water) and organic matter, total N, NH_4_^+^-N, and NO_3_^−^-N of 9.00 g kg^−1^, 0.53 g kg^−1^, 7.81 mg kg^−1^, and 16.47 mg kg^−1^, respectively. Over the past 30 years, the mean annual temperature has been 18.4 °C, the mean annual precipitation has been 1145.5 mm, and the mean annual sunshine has been 1276.7 h. The atmospheric CO_2_ (aCO_2_) concentration in the field was ~415 ppm during the experimental period.

### 4.2. Design and Description of Custom-Built Chambers

The experiment was carried out in 6 identical enclosed gas chambers, which were made of a steel frame structure covered with transparent glass (a thickness of 10 mm and a light transmission rate of 90%) (Yutao Glass Company, Chongqing, China) (length × width × height = 1.5 m × 1.0 m × 2.5 m). The top of the gas chamber was laid with hard plastic tubes, which were evenly perforated. The gas flow solenoid valve (AirTAC (China) Co., Ltd., Yueqing, China) was connected to a metal cylinder containing pure CO_2_ as the gas source. Each chamber was connected to two air pumps (suction and intake), and the excess CO_2_ and water vapor in the chamber were balanced with a 1M NaOH solution and anhydrous CaCl_2_, respectively. A hanging air conditioner (Gree, Zhuhai Gree Company, Zhuhai, China) was installed on the top of the air chamber to regulate the temperature of the room. An atmospheric light, temperature, and humidity sensor (Jingxun Electronic Technology, Weihai, Shandong, China) and a CO_2_ concentration detector (infrared CO_2_ sensor module B-530, ELT SENSOR Corp., Bucheon-si, Gyeonggi-do, Republic of Korea) were installed in the middle of the chamber. All of these devices were deployed using a fully automatic control device (DSS-QZD, Qingdao Shengsen Numerical Control Technology Institute, Qingdao, Shandong, China). The whole system could automatically control a similar temperature, humidity, and CO_2_ concentration inside and outside the glass chamber and ensure that the CO_2_ concentration in the chamber was maintained at the experimental design value [89].

### 4.3. Design of Experiment and Preparation of Materials

In a randomized block design, the experiment consisted of four treatments (two CO_2_ levels and two nitrogen fertilization rates) and each treatment was replicated three times in pots for a total of 12 pots (2 CO_2_ levels × 2 N fertilization rates × 3 replicates for each treatment) (Supplementary Figure S1). Based on the field-detected CO_2_ concentration, we set up two CO_2_ concentration (±30 ppm) treatments: (1) atmospheric CO_2_ (aCO_2_, 410 ppm during daytime/460 ppm at night), and (2) eCO_2_ (eCO_2_, 550 ppm during daytime/610 ppm at night). The time of day and night for the CO_2_ treatment varied with the local sunrise and sunset times and changed with the seasons. The other growth conditions, including a similar light level, temperature, humidity, etc., were automatically adjusted inside and outside the glass growth chamber. A detailed description of the automatically controlled environmental facility used in this study can be found in our previous publications [89,90].

The faba bean (*Vicia faba* L.), an important leguminous crop for supplying plant protein to people, particularly in the countryside, symbiotically fixes N_2_ with rhizobia and, thus, relieves a N deficiency to some degree under eCO_2_ [91]. Faba bean seeds (*V. faba* cv. 89–147) were sterilized with 6% (*v*/*v*) hydrogen peroxide and germinated on sterile filter paper [92]. The germinated seeds were sown in plastic pots (diameter = 22 cm, height = 20 cm, each containing 5 kg of soil) and cultivated until they reached the harvest stage (~5 months old).

Two N fertilization treatments were also applied as (1) no N supply (N0) and (2) 100 mg N kg^−1^ DW soil, in addition to the application of P (100 mg P kg^−1^ DW soil, Ca(H_2_PO_4_)_2_) and K (126 mg K kg^−1^ DW soil, K_2_SO_4_). All the fertilizers were applied and thoroughly mixed before planting the faba beans. The potting preparation, row spacing, N supply, and irrigation followed the common cultivation practices in the local area, and no pesticides or fungicides were used. Four uniform faba bean seedlings were arranged in each pot. To ensure a similar environment for all the plants, the potted plants in the room were rotated once per week. Adequate irrigation was provided to maintain the soil moisture at ~70 ± 5% of the field capacity.

### 4.4. Measurement of Photosynthetic Parameters

During the flowering stage of the faba beans, the photosynthetic parameters were measured. Four to six fully expanded compound leaves, free from visible fungal infections, but possibly with asymptomatic colonization, were carefully selected from the tips of the stems. The measurements were taken between 8:30 and 11:30 a.m. on a sunny morning on 9 April 2019, using a Li-6400XT portable photosynthesis system (Li-Cor Inc., Lincoln, NE, USA) with an internal red–blue light source. The light intensity was set at 1000 μmol m^−2^ s^−1^. The CO_2_ concentration in the reference chamber was maintained at 410 μmol mol^−1^ for the N0 and N100 treatments under aCO_2_, and 550 μmol mol^−1^ for the same treatments under eCO_2_. The recorded parameters included the net photosynthetic rate (Pn), the stomatal conductance (Gs), the intercellular CO_2_ concentration (Ci), and the transpiration rate (E).

### 4.5. Preparation of Plant and Soil Samples

Plant and soil samples were collected at the time of faba bean harvest (5 months old) by wearing disposable gloves to avoid contamination. Any plant material or debris was removed from the soil samples. A total of 10 soil cores from different locations within the same pot were collected to create a composite sample for minimizing spatial variability. The collected samples were packed in sterile ziplocked bags and transported to the laboratory in a portable refrigerator (−18 °C), where they were stored at −80 °C for soil DNA extraction. After removing the residual roots, a portion of the soil samples were ground through 2 mm and 0.25 mm sieves and air-dried for a soil physical and chemical property analysis. The plants were further divided into roots, stems, leaves, and seeds. The plant samples were dried in an oven at 70 °C for 72 h, and then the tissue biomass dry weight, grain yield, and harvest index (ratio of seed biomass/shoot biomass) of the faba beans were determined.

### 4.6. Determination of Plant and Soil Chemical Characteristics

Using a LE438 composite electrode meter (Mettler Toledo Instrument Co., Ltd., Shanghai, China), the soil pH was determined with a soil–water ratio of 1:2.5 (*w*/*v*). The soil organic matter was determined by the K_2_Cr_2_O_7_ external heating method and the soil total N was determined by the Kjeldahl method. The soil soluble inorganic N (NH_4_^+^-N and NO_3_^−^-N) was extracted by the Bremner method. The plant total N was determined using the indophenol blue colorimetric method, which involves boiling the test solution with a mixture of sulfuric acid and hydrogen peroxide, followed by measuring at a wavelength of 690 nm. All these above-mentioned parameters were determined according to the relevant methodologies in [93].

### 4.7. Analysis of Soil Bacterial and Archaeal Community Based on Illumina Sequencing

The total DNA of soil microorganisms was extracted from 2 mL of sludge using a FastDNA^®^ SPIN Kit for Soil (MP Biomedicals, LLC, Irvine, CA, USA). The specific operation was carried out strictly in accordance with the kit’s instructions. The extracted total microbial DNA was stored in a refrigerator at −20 °C for future use. The extracted genomic DNA was detected by 1% agarose gel electrophoresis (Bio-Rad Inc., Hercules, CA, USA) and a NanoDrop-2000 spectrophotometer (NanoDrop Technologies Inc., Wilmington, TX, USA). Three replicates were extracted from each composite soil sample, and the extracted DNA solutions were pooled together. Each treatment had three composite DNA samples. The bacterial community composition of the rhizosphere soil was analyzed by high-throughput amplification sequencing. The forward primer 515FmodF (GTGYCAGCMGCCGCGGTAA) and the reverse primer 806RmodR (GGACTACNVGGGTWTCTAAT) were used for bacterial and archaeal 16S rRNA gene PCR amplification of the V4 region, and sequencing was performed using Illumina MiSeq sequencing technology (Illumina, San Diego, CA, USA). The operational taxonomic units (OTUs) were classified by Usearch (v7.1) with a 97% sequence similarity threshold. The microbial community structure and relative abundance were obtained by OTUs with an online platform, namely the Majorbio Cloud (https://cloud.majorbio.com/, accessed on 10 July 2024).

### 4.8. Statistical Analysis

The data were analyzed using a two-way ANOVA with SPSS 19.0 (SPSS Inc., Chicago, USA) to assess differences under different CO_2_ and N supply levels. The data (means ± SE, *n* = 3) were compared by Duncan’s multiple range test at the *p <* 0.05 level. The statistical analyses were conducted using GraphPad Prism (GraphPad Software, version 8.0.2) to assess the characteristics of the relationships. Alpha diversity analyses were performed using the QIIME2 platform, with the results subsequently visualized through graphical representations generated with Mothur (version 1.30.2; https://mothur.org/wiki/calculators/, accessed on 24 April 2024). A principal component analysis (PCA) was conducted based on the OTU data of each sample using R (version 3.3.1). A redundancy analysis (RDA) was utilized to elucidate the relationships between the sample distributions and environmental factors, with the significance tested via a permutation test akin to an ANOVA, implemented through the vegan package in R (version 3.3.1). A co-occurrence network analysis to uncover interactions among aquatic microorganisms across different groups was conducted using Gephi (version 0.9.2).

## 5. Conclusions

We observed that a N supply and eCO_2_ could improve the plant biomass production of faba beans; however, a N supply still reduced the nodulation of faba beans. The restriction of the N supply impaired the leaf net photosynthesis and CO_2_ assimilation in faba beans. However, the adverse effects of N limitation were ameliorated under eCO_2_, which helped maintain photosynthetic activity. While eCO_2_ led to a decrease in the faba beans’ shoot N and root N, an increase in biomass contributed to a higher proportion of N in the seeds, with a concurrent decrease in the shoots. This redistribution of N could enhance the active N utilization efficiency. Our study also revealed that *Nitrososphaeraceae* exhibited a negative response to a N supply under eCO_2_. On the other hand, a N supply increased the structural complexity of the microbial communities under eCO_2_. These results indicate that an increased N supply is necessary to achieve a higher seed N accumulation and to support a higher complexity of soil microbial communities during faba bean cultivation. Advancements in this area are vital for the sustainable improvement of soil fertility and plant productivity under global environmental change scenarios.

## Data Availability

The data are contained within the article. The data presented in this study are openly available in the [NCBI Sequence Read Archive (SRA) database] at [https://www.ncbi.nlm.nih.gov, accessed on 10 July 2024], reference number [PRJNA1133944].

## Supplementary Materials

The following supporting information can be downloaded at: https://www.mdpi.com/article/10.3390/plants13172483/s1, Table S1: Topological features in co-occurrence networks of different treatments: a fertilized treatment with no N supply (N0) and a N supply (N100) with exposure to atmospheric CO_2_ (aCO_2_) and eCO_2_ (eCO_2_). Figure S1: The design and layout of the experimental treatments. The experiment consisted of four treatments, combining two CO_2_ levels (aCO_2_, daytime/nighttime = 410/460 ppm; eCO_2_, 550/610 ppm) and two N fertilization rates (without N supply—N0—and 100 mg N as urea per kg of soil—N100), and each treatment was replicated three times in pots for a total of 12 pots. Note: three randomly arranged chambers were for either the aCO_2_ or eCO_2_ treatment, while the N0- and N100-fertilized plants were grown together inside the chamber under different CO_2_ treatments. The row spacing between plants inside a pot was 12 cm, while the spacing between pots inside a chamber was 20 cm.
