# Supplementary material for: Impacts of Elevated CO2 and a Nitrogen Supply on the Growth of Faba Beans (Vicia faba L.) and the Nitrogen-Related Soil Bacterial Community
Source: Plants (Basel). 2024 Sep 5;13(17):2483. doi: 10.3390/plants13172483 (PMC11397150; doi:10.3390/plants13172483)
Supplement: Supplementary file 1 [file plants-13-02483-s001.zip › plants-3124410-supplementary.pdf]

Supplementary material

Impacts of Elevated CO_2 and Nitrogen Supply on the Growth of Fababean (*Vicia faba* L.) and Nitrogen-related Soil Bacterial Community

Xingshui Dong[1], Hui Lin[2], Feng Wang[2], Songmei Shi[1], Zhihui Wang[3], Sharifullah Sharifi[1], Junwei Ma[2]*, Xinhua He[1, 4, 5]*

[1] National Base of International S&T Collaboration on Water Environmental Monitoring and Simulation in the Three Gorges Reservoir Region and Centre of Excellence for Soil Biology, College of Resources and Environment, Southwest University, Chongqing 400715, China; xingshuid@outlook.com (X.D.); nsharifullah@gmail.com (S.S.); xinhua.he@uwa.edu.au (X.H.); 2022020@ynau.edu.cn (S.S.)

[2] State Key Laboratory for Managing Biotic and Chemical Threats to the Quality and Safety of Agro-products, Institute of Environment, Resource, Soil and Fertilizers, Zhejiang Academy of Agricultural Sciences, Hangzhou 310021, China; linhui@zaas.ac.cn (H.L.); majw@zaas.ac.cn (J.M.); wangfeng@zaas.ac.cn (F.W.)

[3] State Key Laboratory of Hydraulics and Mountain River Engineering & College of Water Resource and Hydropower, Sichuan University, Chengdu 610065, China; zhihuiwang0928@scu.edu.cn (Z.W.)

[4] Department of Land, Air and Water Resources, University of California at Davis, Davis, CA 90616, USA; huahe@ucdavis.edu (X.H.)

[5] School of Biological Sciences, University of Western Australia, Perth 6009, Australia; xinhua.he@uwa.edu.au (X.H.)

* Correspondence: xinhua.he@uwa.edu.au (X.H.), Tel.: 86-18723289058 and majw@zaas.ac.cn (J.M.), Tel: 86-13857166772

Supplementary Table S1. Topological features in co-occurrence networks of different treatments: a fertilized treatment with no nitrogen (N) supply (N0) and N supply (N100) with exposure to atmospheric CO_2 (aCO_2) and eCO_2 (eCO_2).

	**N0-aCO$_2$**	**N100-aCO$_2$**	**N0-eCO$_2$**	**N100-eCO$_2$**
Edges	187	181	143	163
Negative edges(N)	69	46	49	46
Positive edges(P)	118	135	94	117
P/N ratio	1.710	2.935	1.918	2.543
Nodes	50	50	50	46
Average degree	7.480	7.240	5.720	7.087
Diameter	6	5	7	9
Graph density	0.153	0.148	0.117	0.157
Average clustering coefficient	0.559	0.489	0.511	0.671
Average path length	2.664	2.419	3.237	3.303
Modularity	0.516	0.454	0.541	0.533

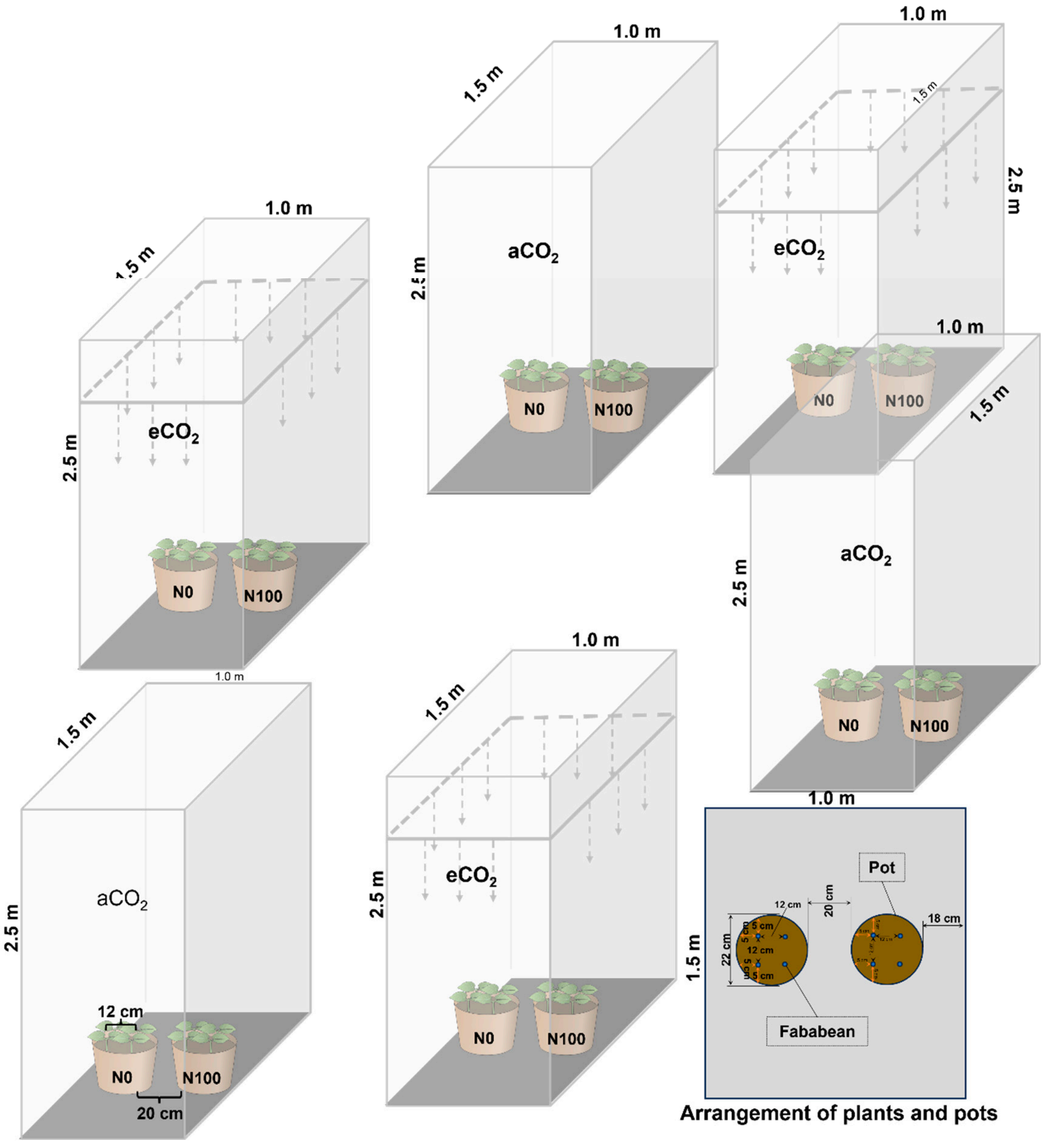

Supplementary Figure S1. Design and layout of the experiment treatments. The experiment consisted of four treatments, combining two CO_2 levels (aCO_2, daytime/nighttime = 410/460 ppm; eCO_2, 550/610 ppm) and two N fertilization rates (without N supply—N0 and 100 mg N as urea per kg of soil—N100), and each treatment was replicated three times or pots for a total of 12 pots. Note: three randomly arranged chambers were for either aCO_2 or eCO_2 treatments, while N0 and N100 fertilized plants grown together inside the chamber under different CO_2 treatments. The row sapcing between plants inside a pot was 12 cm while the spacing between pots inside a chamber was 20 cm.
